# Exploiting *Solanum tuberosum* L. (Vitelotte Noire Cultivar) Peel as a Sustainable Antioxidant Source for Nutraceutical Applications

**DOI:** 10.3390/antiox15050568

**Published:** 2026-04-30

**Authors:** Stefania Peddio, Sonia Lorrai, Alessandra Padiglia, Pierluigi Caboni, Cristina Manis, Antonio Rescigno, Paolo Zucca

**Affiliations:** 1 Department of Biomedical Sciences (DiSB), University Campus, University of Cagliari, 09042 Monserrato, Cagliari, Italy; stefania.peddio@unica.it (S.P.); sonia.lorrai@unica.it (S.L.); pzucca@unica.it (P.Z.); 2 Department of Life and Environmental Sciences (DiSVA), University Campus, University of Cagliari, 09042 Monserrato, Cagliari, Italy; padiglia@unica.it (A.P.); caboni@unica.it (P.C.); cristina.manis@unica.it (C.M.)

**Keywords:** purple potato, antioxidant, by-product

## Abstract

The valorization of agri-food by-products aligns with circular economy principles and offers sustainable sources of bioactive compounds. This study investigated the peels of the purple-fleshed *Solanum tuberosum* L. cv. Vitelotte Noire (VN), cultivated in Sardinia, as a potential resource for nutraceutical antioxidants. Extracts were obtained using solvents of different polarities (water, 80% and 96% ethanol) and characterized. Phytochemical screening revealed high concentrations of total phenolics, flavonoids, and anthocyanins, with the 96% ethanolic extract showing superior anthocyanin content. Antioxidant capacity, assessed via ORAC-PYR, TEAC-ABTS, and DPPH assays, was highest in the alcoholic extracts. Furthermore, all extracts showed protective effects in an in vitro model of AAPH-induced oxidative DNA damage, as indicated by the preservation of plasmid supercoiling. Untargeted LC-QTOF-MS analysis detailed a rich metabolomic profile, including organic acids, amino acids, and vitamins. The findings confirm VN peel as a potent, sustainable source of antioxidants, supporting its valorization for developing high-added-value nutraceutical and functional food ingredients, while reducing waste disposal costs and environmental impact.

## 1. Introduction

In the agri-food sector, substantial quantities of waste biomass are produced every year. Previously designated for disposal or utilized as animal feed or low-value fertilizers, currently these materials are recognized as valuable raw sources for bioactive compounds [1,2]. Within these matrices, a high concentration of phenolic compounds, flavonoids, carotenoids, vitamins, and other natural antioxidants has been reported [3]. For example, fruit peels (such as those of citrus fruits, grapes, or apples) are rich in polyphenols, which are known for their anti-inflammatory and antioxidant properties [4,5]. Similarly, seeds from various fruits, which are often discarded during industrial processing, contain high amounts of essential fatty acids and tocopherols [6,7,8].

Antioxidants are molecules well known for their ability to neutralize free radicals and reactive oxygen species (ROS), responsible for oxidative stress, which is often a key factor in the genesis of several chronic diseases and cellular aging [9]. Primary plant-derived antioxidants include different classes of molecules, such as: polyphenols (e.g., resveratrol, quercetin, catechins), which are particularly abundant in grapes and green tea [10,11]; carotenoids (e.g., β-carotene, lycopene), found in carrots, tomatoes, and bell peppers [12]; and various vitamins (e.g., vitamins C and E), which are prevalent in citrus fruits, kiwis, and dried fruits [13]. The human body already possesses an endogenous antioxidant defense mechanism, but supplementation with exogenous molecules can improve these protective capabilities [14]. Indeed, these products may exert physiological effects and are increasingly gaining relevance in the prevention and treatment of various chronic diseases [15,16].

In this context, the extraction and purification of antioxidant molecules from agricultural waste or food processing by-products not only reduce the environmental impact of waste disposal but also provide added value, promoting a model of circular economy [17]. The extraction of these compounds from agricultural by-products represents a beneficial strategy for environmental sustainability, for cost-effectiveness and for the development of greener extraction processes.

Recent studies have demonstrated that extracts obtained from plant by-products possess antioxidant properties comparable to those of synthetic molecules widely used in the food industry but may pose potential health risks [18,19]. Thus, the valorization of these materials would not only reduce environmental impact but could also pave the way for the development of low-cost dietary supplements or nutraceuticals and functional foods [20].

**Figure 1:**
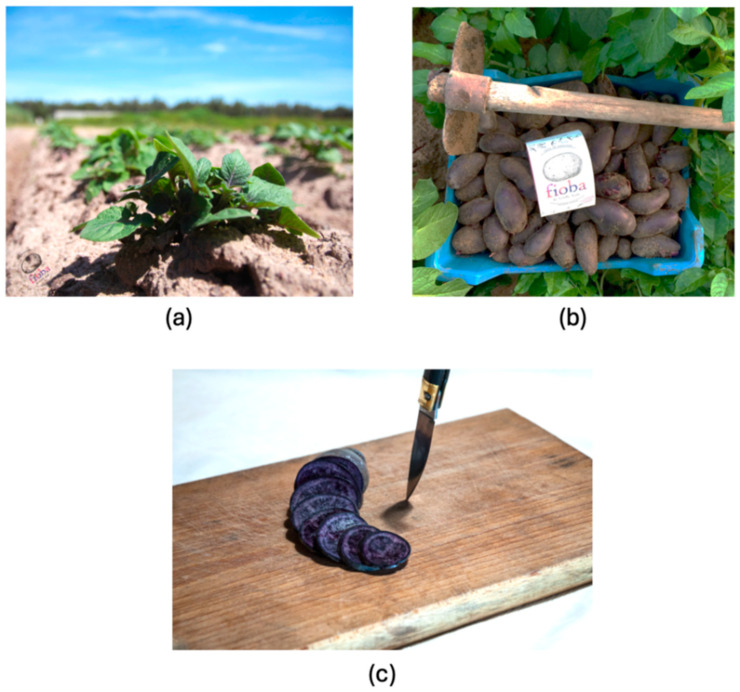
Vitelotte Noire (VN) potato variety object of this study. A cultivated plant (**a**); potatoes harvested (**b**) and cut open to reveal their intensely colored flesh (**c**).

Based on these considerations, this study evaluates the phytochemistry of some extracts from *Solanum tuberosum* L., a variety of Sardinian potatoes belonging to the Solanaceae family. This annual herbaceous plant is cultivated in Sardinia, the second largest island in Italy [21]. This variety of tuber, locally known as Violette Noire (VN), has an elongated, knobby shape and small dimensions. It is characterized by distinctive dark purple coloration of pulp, due to the presence of carotenoids and other antioxidant compounds, as shown in Figure 1 [22,23]. The Mediterranean climate promotes the production of bioactive compounds in many plant species. Plants in Sardinia are subject to significant water stress during the summer season, characterized by long hot periods with very limited rainfall. The deficiency of water and high temperatures stimulates the synthesis of antioxidant substances, involved in cellular defense mechanisms. VN cultivar grown in Sardinia can thus produce a unique and enriched profile of antioxidant compounds in its peel as a defense mechanism.

This study is designed to address the specific knowledge gap regarding the valorization of this agri-food waste product from this geographical area, which has not been previously investigated. Our primary objective is thus to evaluate whether environmental stressors in Sardinia could enhance the antioxidant profile of the VN peel; and second, to identify the most efficient food-grade solvent system for maximizing the recovery of these bioactives for industrial utilization.

To date, few studies have been conducted on the leaves and tubers of different varieties of purple potatoes and their pulp puree to confirm the presence of compounds with antioxidant properties [24,25]. The aim of our study is to analyze the biochemical composition of peels of VN, to assess if a product normally considered waste may contain bioactive compounds. At the same time this study proposes different extraction procedures to maximize the yield of target compounds, assess preliminary antioxidant screening in an in vitro model, and explore possible pilot applications in the formulation of nutraceuticals.

## 2. Materials and Methods

### 2.1. Chemicals

All the reagents were of reagent grade and were used without further purification. Sodium hydroxide hydrochloric acid, potassium chloride, acetic acid, DPPH, TROLOX, iron(III) chloride hexahydrate, ABTS, potassium peroxydisulfate, potassium phosphate monobasic, potassium phosphate dibasic, pyrogallol red and AAPH (2,2′-azobis(2-amidinopropane) dihydrochloride) were purchased from Sigma-Aldrich (St. Louis, MO, USA). Bi-distilled water (<18 MΩ*cm at 25 °C) was obtained with a Milli-Q purification system (Millipore, Milan, Italy).

Ethanol, sodium carbonate monohydrate, aluminum chloratum hexahydrate, Folin–Ciocâlteu’s phenol reagent and 2,4,6-tri(2-pyridyl)-s-triazine were acquired from Merck KGaA (Darmstadt, Germany). The plasmid used for all experiments was pUC18 (Twin Helix, Rho, Italy).

Spectrophotometric measurements were performed using an UltroSpec 2100 pro instrument (Amersham Bioscience, Milan, Italy).

### 2.2. Plant Sample

All materials were purchased from a local Sardinian farm (Fioba—La Violetta Sarda, Arborea, Italy) from September 2024 through December 2024. The underground part of the plants was then gently cleaned. About 20 specimens (around 2 kg) were used to obtain a representative sample. Then the peel was separated from the pulp, frozen to −80 °C within an hour, lyophilized, ground in a knife mill and stored at −20 °C sealed in plastic bags.

### 2.3. Preparation of Solanum tuberosum Extracts for Antioxidant Experiments

Samples of 7.5 g of freeze-dried potato peels were suspended in 99 mL solvent (water or ethanol) and 1 mL formic acid at 70 °C and then stirred at 25 °C for 15 min. The supernatant was collected after centrifugation at 5000× *g* for 10 min. The extraction was repeated 3 times. The whole procedure was repeated using as the solvent water, ethanol 96% and ethanol 80% in water.

The extracts were collected and dehydrated by lyophilization and/or rotary evaporator according to the solvent to be eliminated. The obtained extracts were then stored at −20 °C.

### 2.4. Phenolics Determination

The spectrophotometric Folin–Ciocâlteu approach was used to determinate soluble phenolics content: 2.5 mL of 2% *w*/*v* Na_2_CO_3_ was added to 1 mL of each sample. After 1 min at 25 °C, 0.25 mL 1 N Folin–Ciocâlteu reagent was added. After 45 min of dark incubation at 25 °C the absorbance was measured at 760 nm. Gallic acid was used as the standard (linearity range 0.05–0.6 mM), and the results are expressed as gallic acid equivalents (mGAE), referring both to mg of extract and to mg of dry weight DW.

Spectrophotometric methods were also used for the quantification of total flavonoids and total anthocyanin [26] with minor modifications.

### 2.5. Determination of Antioxidant Capacity

As previously described, the antioxidant capacity was determined using four different spectrophotometric assays [27].

DPPH (1,1-Diphenyl-2-picrylhydrazyl) radical scavenging assay was performed incubating 0.3 mL of sample and 0.7 mL of DPPH solution (25 mg/L in EtOH). After 30 min at 25 °C incubation, the decrease in absorbance at 515 nm was measured and the percentage of DPPH decoloration (%DEC) calculated as follows.

(1) $$\%DEC=100\cdot \frac{Abs_{control}-Abs_{sample}}{Abs_{control}}$$

Trolox was used for the calibration curve (linearity range 5–50 μM), and the results are expressed as Trolox Equivalents (TE) and as IC_50_.

FRAP (Ferric reducing antioxidant power) by incubation of 2.5 mL of 10 mM 2,4,6-tripyridyl-s-triazine (TPTZ) in 40 mM HCl with 25 mL of 0.1 M sodium acetate buffer (pH 3.6) and 2.5 mL of 20 mM FeCl_3_ at 37 °C. Then 0.2 mL of this solution was added to 0.77 mL H_2_O and 0.03 mL of sample. After 6 min at 25 °C, reaction mixtures were centrifuged at 8000× *g* for 10 min, and absorbance at 593 nm measured. The results are expressed as Trolox Equivalents (mTE).

TEAC (Trolox equivalent antioxidant capacity) assay was performed using 2,2′-azinobis(3-ethylbenzothiazoline 6-sulphonate) (ABTS) cationic radical as previously described [27]. The percentage of ABTS decoloration (%DEC) was calculated as shown in Equation (1). The results are expressed as Trolox Equivalents (mTE/g, linearity range 0.1–0.8 mM), and as IC_50_.

In ORAC-PYR (oxygen radical absorbance capacity—pyrogallol red) assay 0.75 mL of 6.6 mM pyrogallol red solution in 75 mM potassium phosphate buffer (pH 7.4) was treated with 0.125 mL of the sample at 25 °C for 10 min. Then 0.125 mL of 0.153 mM 2,2′-azobis(2-amidinopro pane) dihydrochloride (AAPH) solution in 75 mM potassium phosphate buffer (pH 7.4) was added, following the decrease in absorbance at 540 nm for 35 min at 25 °C. The area under the kinetic curve was calculated (AUCnet) by subtracting the area of the blank (AUCblank) from the area of the sample (AUCsample). The results are expressed as Trolox Equivalents (mM TE, linearity range 0.1–0.8 mM).

### 2.6. Plasmid and Reagents

Three antioxidant extracts of plant origin were evaluated: one obtained via aqueous extraction and two prepared using ethanol as the extraction solvent (80% and 96% ethanol). After extraction, each sample was dried using a vacuum centrifuge (Concentrator Plus, Eppendorf, Germany), following the manufacturer’s instructions. The resulting residues were resuspended in nuclease-free water to a final concentration of 50 mg/mL. For all experimental conditions involving plant extracts, 1.5 µL of the resuspension, corresponding to 75 µg of plant-derived material, was used.

Each reaction mixture was assembled on ice in a final volume of 20 µL using TE buffer (1 mM Tris-HCl, 0.1 mM EDTA, pH 8.2) as the diluent, and contained 200 ng of pUC18 plasmid DNA. The following experimental conditions were tested:

1. Control (DNA only): plasmid DNA alone, without any oxidative or antioxidant agent.
2. Oxidative stress condition: DNA incubated with 2 µL of freshly prepared 10 mM AAPH solution.
3. Positive control (Trolox): DNA co-incubated with 2 µL of 10 mM AAPH and 2 µL of 5 mM Trolox.
4. Aqueous plant extract: DNA incubated with 2 µL of 10 mM AAPH and 1.5 µL of the aqueous extract.
5. 80% ethanol extract: DNA incubated with 2 µL of 10 mM AAPH and 1.5 µL of the 80% ethanol-derived extract.
6. 96% ethanol extract: DNA incubated with 2 µL of 10 mM AAPH and 1.5 µL of the 96% ethanol-derived extract.

All reaction mixtures were incubated at 37 °C for 45 min before electrophoretic analysis.

### 2.7. Analysis of DNA Topology by Agarose Gel Electrophoresis

Following incubation, DNA samples were analyzed by agarose gel electrophoresis to assess oxidative damage and topological alterations. Gels were prepared using 1.5% (*w*/*v*) agarose (Euroclone, Pero, Italy) in 1× TBE buffer (pH 8.3) and supplemented with Eurosafe nucleic acid stain (Euroclone). Electrophoresis was carried out at 90 V for 90 min at room temperature. DNA loading was performed by mixing 10 µL of each reaction mixture with 2 µL of 6× loading dye (EURx, Gdańsk, Poland). DNA bands were visualized under UV transillumination using a UVIFORtransilluminator (Elettrofor, Rovigo, Italy) and documented using a digital imaging system equipped with a Moticam 2.0 MP camera (Motic, Xiamen, China).

### 2.8. LC–QTOF-MS Analysis

Based on the mass of each dried extract, samples were reconstituted in methanol by adjusting the solvent volume to reach a final concentration of 1000 mg/L. The reconstituted solutions were then filtered prior to analysis to remove particulate matter. Subsequently, a 50 µL aliquot of each filtered extract was transferred into an autosampler vial and diluted with 950 µL of methanol. Samples were thoroughly mixed before instrumental analysis. All extracts were analyzed using a quadrupole time-of-flight mass spectrometry (QTOF-MS) platform.

Analyses were performed using an Agilent 6560 ion mobility LC/Q-TOF mass spectrometer (Agilent Technologies, Palo Alto, CA, USA) equipped with an electrospray ionization (ESI) source. Injection volumes were set at 4 µL for positive ionization mode and 6 µL for negative ionization mode. Chromatographic separation was achieved on a Kinetex EVO C18 column (5 µm particle size, 100 Å pore size; Kinetex, Torrance, CA, USA). The mobile phases consisted of water containing 0.1 M formic acid (A) and methanol containing 0.1 M formic acid (B). Elution was carried out in gradient mode at a flow rate of 0.4 mL/min as follows: 0–15 min, 0–100% B; 15–19 min, 100% B; 19–21 min, 100–0% B; and 21–24 min, 0% B for column re-equilibration. The ESI source parameters were set as follows: nebulizer pressure 20 psi, drying gas (N_2_) flow rate 5 L/min, and drying gas temperature 325 °C. Data were acquired in both positive and negative ionization modes over a mass range of *m*/*z* 40–1700 to ensure broader metabolome coverage. Instrument control and data processing were performed using MassHunter Qualitative Analysis software (version 10.0, Agilent Technologies).

### 2.9. LC–MS/MS Quantitative Analysis

α-chaconine and α-solanine were measured using chromatographic techniques with a 1260 Agilent Infinity II coupled to an Agilent 6400 triple quadrupole mass spectrometer (Agilent Technologies, Santa Clara, CA, USA). A Kinetex C18 chromatographic column (1.6 μm, 100 mm × 2.1 mm, Phenomenex, Bologna, Italy), maintained at 50 °C, was used for the analysis. The mobile phase consisted of water containing 10 mM ammonium formate (A) and methanol (B), delivered at a flow rate of 0.3 mL/min. The gradient elution started at 5% B, held for 0.2 min, and then linearly increased to 95% B over 3 min. This composition was maintained for 1 min before returning to the initial conditions (5% B) between 4.2 and 5 min. The system was then re-equilibrated for 1 min, followed by an additional 2 min post run to ensure column stabilization before the next injection. Mass spectrometric analysis was conducted using a jetstream (ESI) source in both positive and negative ionization modes. Nitrogen served as the sheath gas, drying gas, and collision gas, with the sheath gas flow rate set at 11 L/min at 375 °C and the drying gas flow rate at 7 L/min at 300 °C. The nebulizer pressure was adjusted to 30 psi, with a capillary voltage of 3500 V and a nozzle voltage of 1500 V. The fragmentor voltage was set to 81 V, and the cell acceleration voltage to 4 V. Data acquisition was performed in multiple reaction monitoring (MRM) mode, controlled by Agilent MassHunter Workstation (Agilent Technologies, Palo Alto, CA, USA). The selected transitions were as follows: α-chaconine (*m*/*z* 852.7 → 97.7, CE = 87 eV) as quantifier and (*m*/*z* 852.7 → 398.4, CE = 73 eV) as qualifier; α-solanine (*m*/*z* 868.9 → 398.6, CE = 81 eV) as quantifier and (*m*/*z* 868.9 → 98.3, CE = 73 eV) as qualifier. The peak area of each quantifier ion was used for quantification after external calibration using authentic standards.

### 2.10. Statistical Analysis

GraFit 7 (Erithacus Software, London, UK), R 2.5.1 (R Foundation for Statistical Computing, Vienna, Austria), and GraphPad Prism 10.2.3 software (GraphPad software, San Diego, CA, USA) were used for data analysis. One-way analysis of variance (ANOVA) and the Bonferroni multiple comparisons test were used to evaluate the statistical significance of the differences.

## 3. Results and Discussion

Recently the scientific community has focused on phytochemicals and plant-based foods. This increasing interest is due to the presence of bioactive compounds well known for their effects against aging, degenerative disorders, and cancer [28]. These molecules act as free radical scavengers, which are known to play a key role in the onset and progression of many diseases.

The *Solanum tuberosum* potatoes of intensely colored varieties, such as the purple VN, are rich sources of various important phytochemical compounds and high levels of natural antioxidants [29].

A significant fraction of these beneficial compounds is concentrated in the potato peel, a portion commonly discarded during industrial processing. In the agri-food sector, especially in the production of potato chips and similar products, the peeling process generates large quantities of waste material, which in turn leads to high disposal costs.

Therefore, the aim of the present study was to verify the phytochemistry and the antioxidant potential in the by-product VN peel (VNP), from the perspective of industrial utilization (e.g., in food, nutraceutical, and cosmetic industries).

### 3.1. Analysis of Phenolics and Antioxidant Capacity

Firstly, the VNP has been extracted using three solvent mixtures at different polarities (water, ethanol 96% and ethanol 80%). All the tested solvents were food grades, industrially viable, and so safe for potential alimentary and nutraceutical preparations. As shown in Table 1, the aqueous extract resulted in one order of magnitude higher extraction yield (15%), whereas the alcoholic and hydroalcoholic extracts gave respectively 2.1 and 1.2% yields. Such different results, combined with our chemical and MS data (see below), suggest water fraction could primarily contain non-phenolic materials like carbohydrates, whereas the lower-yield ethanolic extracts were significantly more concentrated in bioactive compounds such as phenolics and anthocyanins.

The obtained extracts were then resolubilized and subjected to spectrophotometric Folin–Ciocâlteu assay, NaNO_2_/AlCl_3_-based assay, and the differential pH absorbance method for the determination respectively of total phenolics, total flavonoids, and total anthocyanins. The results are summarized in Table 1. Phenolics are more concentrated in alcoholic extracts (*p* < 0.05), and a similar trend is also observed in the case of total flavonoids. When the total contents refer to the dry weight of VNP the aqueous extract presents slightly higher values of phenolics and flavonoids. Taken together, these data are in accordance with the fact that the water mainly extracted chemicals other than phenolics (possibly carbohydrates).

Our data seem higher than other reported data for VN [30]. In this study total phenolics from VN cultivated in Turkey ranged between 1.94 and 1.12 mGAE/g DW. However, the methods of extraction were quite different; for instance, they did not involve organic solvents.

Very similar results have also been recorded with other purple-fleshed potatoes, even when studying only the peels [31,32]. In these studies, it was concluded that purple potatoes on average had higher phenolic contents than classic yellow types.

A study involving variously pigmented potatoes from Romania showed that total polyphenols are influenced by the cultivar’s genetics, crop area, and degree of pigmentation [33].

Previous research has emphasized that the content of phenolic compounds in potatoes is influenced by agrotechnical conditions, the climate, and a degree of ripening during harvest and post-harvest manipulations [34].

Similar results have been obtained for total flavonoids (Table 1), as alcoholic extracts were the most concentrated, ranging between 2.5 and 10.3 mCE/g DW. These values were higher than data reported in the literature, even for the peel [31,33]. However, in this case different methods of extraction, analysis methodology, agrotechnical conditions, climate, and so on should be taken into consideration. In any case, total flavonoids were highest in the purple potato samples [31,33].

Total anthocyanin content was, by contrast, highest in the 96% Et-OH extracts (*p* < 0.05), even higher than the 80% Et-OH sample (Table 1), suggesting a slightly lower polarity for this class of chemicals. A comparison with the reported data in the literature confirms that such compounds are also more concentrated in purple-colored potatoes [33,35].

Total antioxidant activity was then determined using both spectrophotometric electron transfer-based (ET) methods (TEAC-ABTS, FRAP, and DPPH-scavenging) and the hydrogen atom transfer (HAT) method (ORAC-PYR). Both types are essential because they measure different chemical behaviors. ET assays rely on simple reduction, while the HAT assay specifically assesses the ability of phytochemicals to quench radicals via hydrogen atom transfer. This provides a more comprehensive profile of the extract’s antioxidant potential.

The results reported in Table 2 highlight significant differences among the tested assays since the alcoholic extract had the highest activity using ORAC-PYR, TEAC-ABTS and DPPH-scavenging assays. Only using the FRAP method, the aqueous extract performed better, possibly suggesting some specifical chemical in this sample interfering with the specific cupric reduction mechanism of the assay. Our results indicate that VNP has a higher average antioxidant capacity than classic yellow potatoes [31].

Overall, our data seem just slightly lower than those reported for VN from Poland [35]. For instance, in our experiments DPPH-scavenging antioxidant activity range between 3.3 and 10.8 mTE/mg DW, whereas the Polish extract gave about 18.3. However, in this case the whole tuber was used to prepare the extracts, suggesting that in any case a significant part of the bioactive phytochemicals is retained in the peel, being therefore discarded in a major part of the industrial process.

Employing the FRAP method, flour from VN cultivated in Romania gave antioxidant capacity ranging from 16 to 23 mTE/mg DW [30]. Such results are very similar to those for our aqueous extract.

### 3.2. Analysis of Oxidative DNA Damage

**Figure 2:**
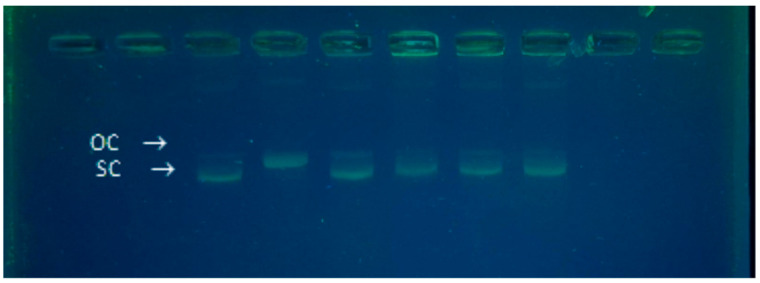
Analysis of plasmid DNA topology by agarose gel electrophoresis following oxidative stress and antioxidant treatments. Representative agarose gel showing the electrophoretic profiles of pUC18 plasmid DNA (150 ng) after incubation under different conditions. Lane 1: untreated control (DNA only); Lane 2: DNA + AAPH (1 mM); Lane 3: DNA + AAPH + Trolox (5 mM); Lane 4: DNA + AAPH + aqueous plant extract; Lane 5: DNA + AAPH + 80% ethanol plant extract; Lane 6: DNA + AAPH + 96% ethanol plant extract. Samples were incubated at 37 °C for 45 min in a final volume of 20 µL and analyzed on a 1.5% agarose gel in 1× TBE buffer containing Eurosafe nucleic acid stain. Electrophoresis was performed at 90 V for 90 min. Migration patterns reflect differences in DNA topology. Under the conditions used, only the supercoiled (SC) and open circular (OC) forms were observed; no linear (L) DNA was detected.

Agarose gel electrophoresis was performed to assess the topological integrity of plasmid DNA following exposure to oxidative stress and antioxidant treatments. As shown in the gel image (Figure 2), distinct differences in plasmid conformation were observed across the six experimental conditions.

In the untreated control sample (lane 1), the plasmid migrated as a single, compact band corresponding to the supercoiled (SC) form. Upon treatment with AAPH (lane 2), the band broadened and shifted upward, indicating the conversion of SC DNA to the open circular (OC) form due to single-strand breaks. Notably, no linear DNA was observed.

When Trolox (lane 3) or plant extracts (lanes 4–6) were added in combination with AAPH, the electrophoretic profiles closely resembled the control, with the preservation of the SC form. This suggests that both Trolox and the tested plant extracts were able to counteract the oxidative stress induced by AAPH and prevent topological relaxation of the plasmid. A similar protective effect of Trolox against oxidative DNA damage has also been demonstrated under UVB-induced stress in vitro [36]. These findings reinforce the utility of plasmid topological analysis as a sensitive approach to detect oxidative DNA damage and antioxidant protection. Importantly, the absence of linear DNA under our conditions is mechanistically justified. AAPH-derived peroxyl radicals typically induce single-strand breaks, leading to SC-to-OC conversion. Double-strand breaks, which result in linear DNA, occur only under more severe conditions. In our study, we deliberately selected mild oxidative conditions, using a 1 mM solution of AAPH and incubating the samples for 45 min at 37 °C without the addition of catalytic metal ions, in order to mimic a more physiologically relevant level of oxidative stress. This experimental design is supported by the existing literature. For example, Hiramoto and coworkers [37] observed the transition from supercoiled to open circular DNA at 1 mM AAPH, while linear DNA was detected only at higher concentrations or with prolonged exposure. Similarly, Paul and colleagues showed that extensive DNA linearization required a radical flux equivalent to at least four radicals per base pair, conditions significantly more aggressive than those used in our study [38]. Spanou and coworkers, using 2.5 mM AAPH, also reported the predominance of open circular forms without clear evidence of linear DNA [39]. Based on this evidence, our choice of conditions was designed to detect subtle topological alterations, such as single-strand breaks, while minimizing background degradation. This approach enhances the biological relevance of the model and allows for a more sensitive assessment of antioxidant protection. Our findings are also consistent with prior reports on plant phenolics. In two different studies, the antioxidant potential of natural extracts using DNA relaxation and radical scavenging assays have been demonstrated [39,40]. Like in those studies, our data show that plant extracts preserved the SC form under oxidative stress, confirming their protective efficacy.

In summary, the DNA topology changes observed in our study are mechanistically consistent with AAPH-induced oxidation, and the protective effects of Trolox and plant-derived antioxidants are in line with evidence from the literature. The absence of linear DNA reflects the mild, physiologically relevant conditions adopted, which favor single-strand breaks as an early indicator of oxidative damage.

Although the electrophoretic profiles clearly indicate differences among experimental conditions, this analysis should be considered qualitative, as no densitometric quantification of DNA forms was performed. Therefore, the results are interpreted as indicative of relative protective effects rather than absolute quantitative differences. The observed patterns were consistent across independent experiments.

This study demonstrates the value of plasmid DNA topology as a sensitive and practical readout for monitoring oxidative DNA damage and evaluating the protective potential of antioxidant compounds. By employing moderate experimental conditions that emulate physiologically relevant oxidative stress, we were able to distinguish subtle topological changes in the absence of extensive degradation. The preservation of the supercoiled conformation in the presence of Trolox and plant-derived extracts underscores their effectiveness in limiting peroxyl radical-mediated strand scission.

### 3.3. LC–QTOF-MS Analysis

The metabolite profile analysis of VN potato peel extracts reveals a diverse array of bioactive compounds, including organic acids, amino acids, and other phytochemicals, with different concentrations based on the solvent used for extraction. Metabolite annotation was performed according to the Metabolomics Standards Initiative (MSI) guidelines. Compound identification was based on high-resolution accurate mass measurements and MS/MS fragmentation patterns, supported by comparison with the literature and spectral databases. As no authentic standards were used, all metabolites were annotated at MSI Level 2 (putatively identified compounds). The full list of annotated metabolites is reported in Table 3.

Ethanol 96% extracts showed higher levels of organic acids like malate and citrate, which are integral to cellular energy metabolism and may confer health benefits. These findings align with studies emphasizing the nutritional value of purple-fleshed potatoes due to their high content of beneficial compounds [21]. The 96% EtOH extract showed the highest levels for metabolites putatively annotated as malate, citrate, succinate, fumarate and α-Ketoglutarate, suggesting that concentrated ethanol favored the recovery of several abundant organic acids which are well-recognized constituents of *Solanum tuberosum* polar extracts and are routinely detected together with amino acids and other polar/semi-polar metabolites in LC-based profiling studies [41]. Furthermore, several nitrogen-containing metabolites of nutritional and physiological relevance, including choline, nicotinamide, GABA, and putrescine, were comparatively higher in the 96% EtOH extract. This pattern suggests that concentrated ethanol may favor the extraction of compounds related not only to central metabolism but also to membrane metabolism (choline) and NAD/NADP biochemistry (nicotinamide). These metabolites are commonly reported in plant-derived matrices and support the potential of potato peel by-products as a source of added-value small molecules beyond phenolics and anthocyanins [42]. The amino acids and related metabolites detected in VN peel extracts exhibited a clear solvent-dependent distribution that closely reflects the experimental data. In particular, phenylalanine and tyrosine were markedly enriched in the 96% ethanol extract, with phenylalanine reaching ~358,731 compared to ~258,642 in 80% ethanol and ~108,038 in water, while tyrosine showed an even stronger shift (561,172 vs. 403,354 and only ~29,023 in the aqueous extract). A similar trend was observed for asparagine (~95,288 in 96% EtOH vs. ~57,120 in water), suggesting that less polar hydroalcoholic systems improved the recovery of several semi-polar nitrogen-containing metabolites. Conversely, tryptophan displayed more comparable intensities across solvents, indicating a lower sensitivity to polarity under the adopted extraction conditions, whereas trigonelline was slightly higher in the aqueous extract, consistent with its strong polarity and zwitterionic character. Overall, this pattern supports the interpretation that the metabolite profile of potato matrices is strongly influenced by extraction parameters, as amino acids and organic acids are known to be major constituents of potato tubers and peels, but their measured abundance depends on solvent composition and matrix–solvent interactions. This behavior has been widely reported in metabolomic studies of potato, where extraction strategy significantly affects the recovery of primary metabolites and thus the observed biochemical fingerprint [41].

The peel of VN potatoes is a rich source of diverse bioactive compounds, including phenolic acids, chlorogenic acid isomers, flavonoids, glycoalkaloids, and vitamins, each contributing to the antioxidant and health-promoting properties of the peel. Phenolic acids such as quinate, shikimate, vanillic, caffeic, p-coumaric, and ferulic acids are integral to the phenylpropanoid pathway in plants, playing roles in defense mechanisms and structural integrity. These compounds exhibit antioxidant activities, aiding in the neutralization of free radicals and potentially reducing the risk of chronic diseases [43]. Among the compounds annotated at MS/MS level (MSI Level 2), quinic acid and its conjugates emerged as major constituents, with quinate reaching its highest intensity in the 96% ethanol extract (~7.45 × 10^6^), markedly exceeding both 80% ethanol and water. This observation is particularly relevant because quinic acid acts as a key precursor in the biosynthesis of chlorogenic acids through the shikimate–phenylpropanoid pathway, which is highly active in potato tissues [44]. Consistently, chlorogenic acid was one of the most abundant phenolics, showing very high signals across all solvents and peaking again in 96% ethanol (~3.42 × 10^7^). Its positional isomers, such as neochlorogenic (3-CQA) and cryptochlorogenic acid (4-CQA), followed the same trend, with cryptochlorogenic acid exceeding 6.9 × 10^6^ in the most concentrated ethanol extract, suggesting improved recovery of hydroxycinnamate esters under reduced polarity conditions. Chlorogenic acids are widely recognized as the predominant phenolics in potato peel and are major contributors to antioxidant capacity and potential health benefits [44].

A similar solvent-driven enrichment was observed for hydroxycinnamic acids, particularly caffeic and ferulic acids. Caffeic acid reached extremely high intensities (~4.03 × 10^7^ in 96% EtOH), nearly sixfold higher than in the aqueous extract, while ferulic acid exceeded 4.16 × 10^6^ under the same conditions. These compounds are well known for their radical scavenging activity and for reinforcing plant structural defense, and their abundance supports the view that potato peel is a valuable source of functional phenolics [45]. Interestingly, shikimic acid increased sharply with ethanol concentration, suggesting efficient extraction of upstream intermediates of aromatic amino acid biosynthesis, whereas vanillic and p-coumaric acids showed more moderate but still solvent-dependent increases.

Beyond phenolics, the dataset highlights the remarkable accumulation of steroidal glycoalkaloids, especially α-chaconine, which displayed the highest overall intensity among all detected metabolites (>1.4 × 10^8^ in 96% EtOH). Together with α-solanine and solanidine, these compounds are predominantly localized in the peel rather than the flesh, where they function as natural defense molecules against pathogens and herbivores.

From a toxicological perspective, α-solanine and α-chaconine are biologically active compounds that can exert adverse effects in humans. Their toxicity is mainly associated with membrane disruption and inhibition of acetylcholinesterase activity, leading to gastrointestinal and neurological symptoms such as nausea, vomiting, diarrhea, and, in severe cases, neurological impairment [46]. Acute intoxication events linked to elevated glycoalkaloid levels in potatoes have been documented, highlighting their relevance for food safety assessments [47]. For this reason, their presence must be carefully considered when evaluating potential applications; accordingly, a targeted LC–MS/MS quantitative analysis was conducted to ensure accurate determination of α-chaconine and α-solanine levels, especially considering the prospective use of this peel as a nutraceutical ingredient. As shown in Table 4, the quantitative results clearly demonstrated that the extraction solvent significantly affected glycoalkaloid recovery. The 96% ethanolic extract showed the highest concentrations, with α-chaconine at 220.29 ± 49.10 mg/kg and α-solanine at 361.55 ± 26.80 mg/kg. Lower but still substantial levels were observed in the 80% ethanolic extract, with α-chaconine at 157.43 ± 29.04 mg/kg and α-solanine at 180.24 ± 36.20 mg/kg. In contrast, the aqueous extract contained markedly reduced amounts, with α-chaconine at 15.03 ± 5.79 mg/kg and α-solanine at 27.04 ± 8.56 mg/kg. This trend is consistent with the semi-polar nature of glycoalkaloids, which are more efficiently extracted in hydroalcoholic systems, particularly at higher ethanol concentrations [48]. The increased recovery observed in 96% ethanol suggests that less polar extraction environments favor the solubilization of these steroidal glycoalkaloids, whereas water alone is significantly less effective.

It is generally accepted that total glycoalkaloid levels exceeding 20 mg/100 g fresh weight (200 mg/kg) may pose a risk to human health [49]. Although the concentrations reported in this study refer to extracts rather than fresh plant material, the relatively high levels detected in ethanolic extracts, particularly in the 96% ethanol system, highlight the importance of dose control in downstream applications. Conversely, the significantly lower glycoalkaloid content observed in aqueous extracts suggests that such systems may represent a safer alternative when minimizing toxicological risk is a priority.

While glycoalkaloids can pose toxicological risks at high concentrations, their documented antimicrobial and cytotoxic properties highlight a potential added value, provided that their levels are strictly controlled [49]. In this context, the quantitative determination performed in the present study is essential to define a safe range of application, particularly considering the potential use of the peel as a nutraceutical ingredient.

Flavonoids were detected at lower absolute intensities but still exhibited meaningful solvent effects. Quercetin and rutin were substantially higher in the 96% ethanol extract, while isoquercitrin, dicaffeoylquinic acid and adenosine were not detected in the aqueous extract, suggesting that less polar solvent systems enhance the extraction of flavonol glycosides and related metabolites. The lack of detectable levels of adenosine, isoquercitrin, and dicaffeoylquinic acid in water extracts, compared to their presence in hydroalcoholic and ethanolic extracts, is likely related to differences in extraction efficiency associated with solvent composition. Although these compounds exhibit moderate to high polarity, hydroalcoholic systems are known to enhance the recovery of a wider range of metabolites by improving matrix penetration and reducing interactions with cell wall components [50]. It should also be noted that the lack of detectable signal in aqueous extracts likely reflects concentrations below the detection limit of the analytical method rather than a true absence of these compounds.

Colored potato varieties are known to accumulate flavonoids that contribute synergistically to antioxidant activity alongside phenolic acids [51]. Additionally, the detection of pyridoxine (vitamin B6) and nicotinic acid (niacin) further enhances the nutritional relevance of the peel, as potatoes are recognized dietary sources of B vitamins involved in amino acid metabolism and redox reactions.

Overall, data strongly support the concept that potato peels are not merely an industrial waste, but a reservoir of structurally diverse phytochemicals whose extractability is tightly governed by solvent polarity. The superior recovery observed with 96% ethanol suggests that moderately polar organic solvents may better disrupt matrix–metabolite interactions and solubilize phenylpropanoid derivatives, a behavior frequently reported in plant metabolomics. These findings align with current strategies aimed at the sustainable valorization of potato by-products for functional food, nutraceutical, or cosmetic applications, where tailoring extraction conditions is essential to maximize the yield of targeted bioactives.

These findings should be interpreted as indicative of chemical antioxidant potential under controlled in vitro conditions, and further biological validation will be necessary to confirm their relevance in complex biological systems.

## 4. Conclusions

This study confirmed that the peels of the tuber *Solanum tuberosum* L. cv. Vitelotte Noire (VN), a by-product of the agri-food industry, could possibly represent a sustainable source of antioxidant compounds with potential applications in several food sectors.

VN peel contains total phenols, flavonoids, and anthocyanins, with the 96% ethanolic extract having the highest anthocyanin concentration and the best overall antioxidant activity, as evidenced by ORAC-PYR, TEAC-ABTS, and DPPH assays, and LC-MS analysis. The protective effect observed in the plasmid DNA assay supports the ability of these extracts to counteract peroxyl radical-induced oxidative damage under controlled experimental conditions, preserving DNA topology and limiting single-strand breaks. In this context, the plasmid-based system provides a sensitive and reproducible model to assess direct antioxidant activity at the molecular level. However, it is important to underline that this experimental approach represents a simplified in vitro system and does not fully account for the complexity of biological environments, including factors such as bioavailability, cellular uptake, metabolism, and interaction with endogenous antioxidant systems. Therefore, the results obtained from this model should be interpreted as preliminary and indicative of antioxidant potential.

LC-QTOF-MS analysis provided a detailed profile of the chemical composition, identifying and quantifying a wide range of bioactive metabolites, including organic acids (e.g., malate, citrate), amino acids (e.g., phenylalanine, tryptophan), vitamins (ascorbic acid), and other molecules of nutraceutical interest such as choline and trigonelline.

In conclusion, this work supports the valorization of VN peels within the framework of a biologically based circular economy, which aims to recycle and reintegrate agricultural waste into the market with added value, contributing to a reduction in disposal costs and waste. Such comprehensive extract characterization can be a preliminary essential prerequisite for future bioactivity-guided fractionation studies.

Extracting antioxidants from this by-product would not only reduce its environmental impact and disposal costs, but also generate low-cost, high-value raw materials for the development of supplements and functional ingredients. However, future studies are needed to optimize extraction processes on an industrial scale, evaluate the stability and bioavailability of active ingredients in complex matrices, and confirm biological efficacy through more advanced cellular and preclinical models.

## Figures and Tables

**Table 1:** Total phenolic, total flavonoid and total anthocyanin content of the extracts from VN peels.

		H_2_O	Et-OH 96%	Et-OH 80%
Yield	%	15 ± 3	2.1 ± 0.4	1.2 ± 0.3
Total phenolics	(mGAE/mg extract)	82 ± 1	292 ± 1	203 ± 1
	(mGAE/mg DW)	12.3 ± 0.2	7.3 ± 0.3	2.4 ± 0.2
Total flavonoids	(mCE/mg extract)	69 ± 1	360 ± 12	205 ± 1
	(mCE/g DW)	10.3 ± 0.2	7.6 ± 0.3	2.5 ± 0.1
Total anthocyanins	μg cyanindin 3-O-glucoside/g extract	1.4 ± 0.1	19.5 ± 0.1	5.8 ± 0.1
	μg cyanindin 3-O-glucoside/mg DW	210 ± 3	409.5 ± 2.1	69.6 ± 1.2

Data are the means of at least three independent determinations ± SD.

**Table 2:** Total antioxidant capacity of the extracts from VN peels.

		H_2_O	Et-OH 96%	Et-OH 80%
ORAC-PYR	(mTE/mg extract)	134 ± 41	235 ± 73	281 ± 54
	(mTE/mg DW)	20.1 ± 6.2	4.9 ± 1.5	3.4 ± 0.6
DPPH	(mTE/mg extract)	72 ± 3	620 ± 12	278 ± 3
	(mTE/mg DW)	10.8 ± 0.5	13.0 ± 0.3	3.34 ± 0.04
	IC_50_ (μg/mL)	51 ± 7	22 ± 5	15 ± 4
TEAC-ABTS	(mTE/mg extract)	137 ± 4	313 ± 11	250 ± 16
	(mTE/mg DW)	20.6 ± 0.6	6.6 ± 0.2	3.0 ± 0.2
	IC_50_ (μg/mL)	105 ± 24	42 ± 9	58 ± 7
FRAP	(mTE/mg extract)	109 ± 2	21 ± 1	36 ± 1
	(mTE/mg DW)	16.4 ± 0.3	0.44 ± 0.02	0.43 ± 0.01

Data are the means of at least three independent determinations ± SD.

**Table 3:** Untargeted LC–MS metabolomic characterization of Vitelotte Noire potato peel extracts highlighting the solvent-dependent distribution. Signal intensities are reported as mean ± SD.

Class	Compound	Main Role	Formula	*m*/*z*	Adduct	Et-OH 80%	H_2_O	Et-OH 96%
Organic acids	Malate	TCA cycle	C_4_H_6_O_5_	133.0142	[M − H]^−^	590,729.38 ± 80,040.65	492,282.88 ± 50,046.88	1,663,659.34 ± 990,408.50
Organic acids	Citrate	TCA cycle	C_6_H_8_O_7_	191.0197	[M − H]^−^	159,019.64 ± 48,736.58	75,182.02 ± 49,936.86	978,547.12 ± 584,453.72
Organic acids	Succinate	TCA cycle	C_4_H_6_O_4_	117.0193	[M − H]^−^	32,389.88 ± 4671.95	25,809.18 ± 339.79	46,968.34 ± 2284.65
Organic acids	Fumarate	TCA cycle	C_4_H_4_O_4_	115.0036	[M − H]^−^	64,938.04 ± 8754.41	71,957.50 ± 7521.81	121,897.05 ± 15,582.95
Organic acids	α-Ketoglutarate	TCA cycle	C_5_H_6_O_5_	145.0142	[M − H]^−^	1989.39 ± 598.46	1766.66 ± 483.66	2530.91 ± 649.04
Organic acids	Quinate	Phenolic precursor	C_7_H_12_O_6_	191.0561	[M − H]^−^	3,162,971.91 ± 743,469.34	2,894,883.42 ± 423,014.42	7,451,298.01 ± 2,150,612.73
Organic acids	Shikimate	Phenolic pathway	C_7_H_10_O_5_	173.0455	[M − H]^−^	15,092.64 ± 11,807.90	70,228.46 ± 14,297.16	151,551.77 ± 54,443.91
Nucleosides	Adenosine	Nucleotide metabolism	C_10_H_13_N_5_O_4_	268.104	[M + H]^+^	106,606.57 ± 26,182.94	0.00 ± 0.00	235,249.40 ± 57,267.32
Amino acids	Asparagine	Nitrogen metabolism	C_4_H_8_N_2_O_3_	133.0608	[M + H]^+^	62,367.60 ± 15,426.84	57,119.68 ± 8083.63	95,288.40 ± 15,220.98
Amino acids	Phenylalanine	Phenolic precursor	C_9_H_11_NO_2_	188.0682	[M + Na]^+^	258,642.07 ± 49,397.03	108,038.46 ± 32,286.79	358,730.54 ± 22,050.67
Amino acids	Tyrosine	Phenolic precursor	C_9_H_11_NO_3_	182.0812	[M + H]^+^	403,354.45 ± 266,065.71	29,022.54 ± 24,223.29	561,171.65 ± 5148.22
Amino acids	Tryptophan	Secondary metabolism	C_11_H_12_N_2_O_2_	205.0972	[M + H]^+^	36,468.43 ± 8095.22	40,927.78 ± 11,682.88	61,123.22 ± 8768.85
Amino acids	GABA	Stress response	C_4_H_9_NO_2_	104.0706	[M + H]^+^	138,757.62 ± 41,557.60	106,397.88 ± 23,378.21	305,101.51 ± 31,175.32
Amino acid derivatives	Trigonelline	Bioactive alkaloid/antioxidant-related	C_7_H_7_NO_2_	138.055	[M + H]	323,785.58 ± 32,124.25	412,339.74 ± 15,641.15	403,970.51 ± 73,412.41
Amines	Putrescine	Cell growth	C_4_H_12_N_2_	89.1073	[M + H]^+^	165,094.45 ± 82,008.26	86,497.97 ± 66,113.47	245,458.78 ± 15,497.99
Amines	Choline	Membrane metabolism	C_5_H_14_NO	105.1148	[M + H]^+^	861,017.27 ± 20,737.70	656,056.60 ± 42,071.97	984,762.72 ± 77,460.85
Vitamins	Nicotinamide	Cofactor (B3)	C_6_H_6_N_2_O	123.0553	[M + H]^+^	363,490.39 ± 57,746.26	319,073.52 ± 64,031.15	606,295.64 ± 29,498.40
Vitamins	Nicotinic acid	Vitamin B3	C_6_H_5_NO_2_	124.0393	[M + H]^+^	99,737.55 ± 19,967.38	129,345.92 ± 6463.35	155,073.41 ± 777.75
Vitamins	Pyridoxine	Vitamin B6	C_8_H_11_NO_3_	170.0812	[M + H]^+^	85,504.64 ± 11,146.49	123,149 ± 7968.38	158,810.33 ± 55,360.17
Vitamins	Ascorbic acid	Antioxidant	C_6_H_8_O_6_	177.0394	[M + H]^+^	3775.06 ± 1486.91	1931.40 ± 3345.29	6076.18 ± 1303.27
Phenolic acids	Caffeic acid	Antioxidant	C_9_H_8_O_4_	179.035	[M − H]^−^	18,775,817.45 ± 5,780,814.04	6,185,667.80 ± 1,080,059.67	40,292,198.19 ± 5,262,388.64
Phenolic acids	Chlorogenic acid	Major antioxidant	C_16_H_18_O_9_	353.0878	[M − H]^−^	18,927,939.97 ± 6,010,546.94	9,347,666.35 ± 783,262.70	34,159,447.62 ± 12,814,998.86
Phenolic acids	Ferulic acid	Antioxidant	C_10_H_10_O_4_	193.0506	[M − H]^−^	2,260,604.50 ± 558,851.51	601,507.35 ± 52,452.60	4,165,725.36 ± 1,799,385.57
Phenolic acids	p-Coumaric acid	Antioxidant	C_9_H_8_O_3_	163.04	[M − H]^−^	277,780.87 ± 79,997.90	83,511.43 ± 16,998.01	507,220.93 ± 68,981.93
Phenolic acids	Vanillic acid	Antioxidant	C_8_H_8_O_4_	167.035	[M − H]^−^	50,857.97 ± 17,650.10	38,357.31 ± 9805.89	70,061.32 ± 1930.67
Phenolic acids	Neochlorogenic acid (3-CQA)	Antioxidant	C_16_H_18_O_9_	377.0843	[M + Na]^+^	453,746.11 ± 272,634.97	149,900.89 ± 68,049.23	3,356,015.05 ± 644,990.26
Phenolic acids	Cryptochlorogenic acid (4-CQA)	Antioxidant	C_16_H_18_O_9_	377.0843	[M + Na]^+^	2,190,731.76 ± 1,157,205.99	467,614.55 ± 199,712.63	6,948,102.18 ± 1,372,572.24
Phenolic acids	Feruloylquinic acid	Antioxidant/phenolic derivative	C_17_H_20_O_9_	391.0999	[M + Na]^+^	440,606.12 ± 319,345.74	181,236.20 ± 42,549.62	1,675,373.49 ± 100,961.12
Phenolic acids	Caffeoylshikimic acid	Antioxidant/phenolic derivative	C_16_H_16_O_8_	337.0918	[M + H]^+^	1,334,439.03 ± 36,376.75	610,718.56 ± 89,947.31	1,402,773.13 ± 173,583.34
Phenolic acids	Dicaffeoylquinic acid	Antioxidant/phenolic derivative	C_25_H_24_O_12_	517.134	[M + H]^+^	63,620.66 ± 35,581.99	0.00 ± 0.00	51,193.00 ± 6337.76
Flavonoids	Quercetin	Antioxidant	C_15_H_10_O_7_	303.05	[M + H]^+^	84,316.25 ± 46,340.63	38,154.46 ± 8580.26	210,283.96 ± 2801.12
Flavonoids	Quercetin-3-O-glucoside (isoquercitrin)	Antioxidant	C_21_H_20_O_12_	487.0847	[M + Na]^+^	14,395.96 ± 4113.87	0.00 ± 0.00	10,980.07 ± 992.32
Flavonoids	Rutin	Antioxidant	C_27_H_30_O_16_	609.1461	[M − H]^−^	40,917.77 ± 21,752.53	16,202.29 ± 2922.89	84,985.48 ± 31,857.37
Glycoalkaloids	Solanidine	Bioactive	C_27_H_43_NO	398.3417	[M + H]^+^	24,354,894.69 ± 17,921,476.20	34,808,776.29 ± 1,385,219.88	32,102,221.39 ± 7,187,307.11
Glycoalkaloids	α-Chaconine	Glycoalkaloid	C_45_H_73_NO_14_	852.5104	[M + H]^+^	131,626,275.25 ± 9,348,650.26	99,852,578.75 ± 6,316,231.85	141,637,090.51 ± 5,235,477.35
Glycoalkaloids	α-Solanine	Glycoalkaloid	C_45_H_73_NO_15_	868.5053	[M + H]^+^	5,901,311.27 ± 1,442,090.28	2,234,128.22 ± 452,032.66	11,157,863.88 ± 1,302,566.65
Fatty acids	Palmitic acid	Lipid metabolism	C_16_H_32_O_2_	255.2329	[M − H]^−^	9,624,871.39 ± 831,114.67	3,045,413.53 ± 663,084.52	13,823,965.05 ± 1,803,586.82
Fatty acids	Oleic acid	Bioactive lipid	C_18_H_34_O_2_	281.2486	[M − H]^−^	6,210,077.38 ± 1,360,691.96	1,243,838.14 ± 140,079.57	8,487,971.73 ± 6,839,412.37
Fatty acids	Linoleic acid	Bioactive lipid	C_18_H_36_O_2_	279.2329	[M − H]^−^	25,992,553.93 ± 4,588,279.48	541,977.46 ± 124,806.81	29,948,372.02 ± 21,387,190.21
Fatty acids	Linolenic acid	Bioactive lipid	C_18_H_30_O_2_	277.2173	[M − H]^−^	12,503,859.85 ± 2,796,760.52	108,833.94 ± 36,663.26	14,893,970.92 ± 11,262,047.01

**Table 4:** Quantitative analysis of α-solanine and α-chaconine by LC-MS/MS.

Glycoalkaloid	96% EtOH Extract (mg/kg)	80% EtOH Extract (mg/kg)	Water Extract (mg/kg)
α-chaconine	220.29 ± 49.10	157.43 ± 29.04	15.03 ± 5.79
α-solanine	361.55 ± 26.80	180.24 ± 36.20	27.04 ± 8.56

## Data Availability

The original contributions presented in this study are included in the article. Further inquiries can be directed to the corresponding author.

## Abbreviations

The following abbreviations are used in this manuscript:

AAPH	2,2′-azobis(2-amidinopropane) dihydrochloride
ABTS	2,2′-azinobis(3-ethylbenzothiazoline 6-sulphonate)
DPPH	1,1-Diphenyl-2-picrylhydrazyl
DW	Dry Weight
FRAP	Ferric Reducing Antioxidant Power
ORAC-PYR	Oxygen Radical Absorbance Capacity—Pyrogallol Red
SC/OC	Supercoiled/Open Circular
TEAC	Trolox Equivalent Antioxidant Capacity
VN	Vitelotte Noire
